# Prevalence and serogroup changes of *Neisseria meningitidis* in South Korea, 2010–2016

**DOI:** 10.1038/s41598-018-23365-8

**Published:** 2018-03-28

**Authors:** Hyukmin Lee, Younghee Seo, Kyung-Hyo Kim, Kyungwon Lee, Kang-Won Choe

**Affiliations:** 1 Department of Laboratory Medicine and Research Institute of Bacterial Resistance, Yonsei University College of Medicine, Seoul, 03722 South Korea; 2 Department of Pediatrics and Center for Vaccine Evaluation and Study, Medical Research Institute, Ewha Womans University School, Seoul, 07985 South Korea; 3 Department of Internal Medicine, Armed Forces Medical Command, Seongnam, 13574 South Korea

## Abstract

Determination of the major serogroups is an important step for establishing a vaccine programme and management strategy targeting *Neisseria meningitidis*. From April 2010 to November 2016, a total of 25 *N. meningitidis* isolates were collected in South Korea, in collaboration with the Korean Society of Clinical Microbiology. Among isolates, 19 isolates were recovered from blood and/or cerebrospinal fluid (CSF) in 46 patients who suffered from invasive meningococcal disease (IMD), and six isolates were found in sputum or the throat. The most common serogroup was serogroup B (overall, 36%, n = 9/25; IMD, 37%, n = 7/19), which was isolated in every year of the research period except for 2011. There were five serogroup W isolates recovered from patients in military service. W was no longer isolated after initiation of a vaccine programme for military trainees, but serogroup B caused meningitis in an army recruit training centre in 2015. In MLST analysis, 14 sequence types were found, and all isolates belonging to W showed the same molecular epidemiologic characteristics (W:P1.5-1, 2-2:F3-9:ST-8912). All isolates showed susceptibility to ceftriaxone, meropenem, ciprofloxacin, minocycline, and rifampin; however, the susceptibility rates to penicillin and ampicillin for isolates with W and C capsules were 22% and 30%, respectively.

## Introduction

*Neisseria meningitidis* isolates can cause asymptomatic colonization or severe invasive infections. Invasive meningococcal disease (IMD) may develop as acute sepsis or meningitis^1^, and meningococcal meningitis combined with septic shock is responsible for a higher mortality (adjusted odds ratio, 23.3) than simple meningitis^2^. Although infants and children are the main targets of *N. meningitidis* infection, outbreaks in adolescents and young adults can occur as well. The prevalence is diverse in different countries, ranging from 0.16-1.65 cases/100,000 individuals in well-developed countries to over 300 cases/100,000 individuals in the sub-Saharan meningitis belt^1,3^. According to global disability-adjusted life years (DALY) estimation, the burdens of total bacterial and meningococcal meningitis were 21,014.9 and 4,314.7 (20.5% of the total bacterial meningitis burden) in 2013, repectively^4^.

The clinical manifestations of meningococcal meningitis can vary and include headache and neck stiffness; some patients may be misdiagnosed at early stages due to vague symptoms. Meningitis combined with meningococcal septic shock can result in death in approximately 30% of patients, with an increasing death rate associated with age (adjusted odds ratio of 1.02 per 1-year increase in age)^5^. Rapid progression and high mortality can make it difficult to properly manage patients in certain circumstances, although vaccines have been developed to overcome this serious infection. The most important virulence factor causing invasive infection is the polysaccharide capsule, and this structure is the main target of vaccines. Currently, 12 different serogroups of *N. meningitidis* can be distinguished^5,6^. Determination of the major serogroups is important in establishing a vaccine programme and management strategy because the distribution of prevalent serogroups may be diverse based on the country and other factors. On the other hand, an IMD outbreak can also occur among young adults who reside in dormitories. Because of the high mortality and morbidity of invasive meningococcal infections, appropriate molecular epidemiologic analysis is required to manage and control dissemination. The recent emergence of antimicrobial resistance to penicillin has become another problem for managing and treating *N. meningitidis* infections^7^. In this study, we aimed to investigate the sero/genogroups, PorA subtypes, FetA subtypes, multilocus sequence types (MLSTs), and antimicrobial susceptibility of *N. meningitidis* using isolates collected across South Korea.

## Results

**Figure 1:**
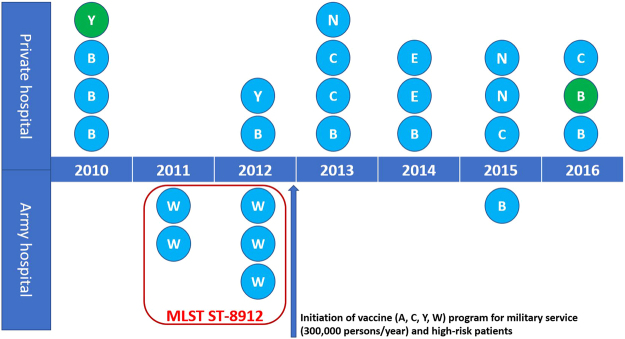
Summary of the number of isolates collected and their serogroups from 2010 to 2016 in South Korea according to patient type. Each letter designates the serogroups for each isolate: B, serogroup B; C, serogroup C; W, serogroup W; Y, serogroup Y; E, serogroup E; N, non-typeable serogroup. The green colour indicates a carrier (throat).

From April 2010 to November 2016, a total of 46 IMD patients who could represent all cases of IMD in Korea were reported to the Korea Centers for Disease Control and Prevention (CDC), and 19 isolates were recovered from blood and/or cerebrospinal fluid (CSF) among 46 IMD patients. Four isolates were recovered from the sputum of patients who were admitted under suspicion of pneumonia, and two isolates that could be regarded as normal colonizers were incidentally recovered from the throat. All isolates were collected from 16 laboratories, which were located distantly in South Korea, and the number of isolates ranged broadly from two to five isolates per year (Fig. 1). The ages of the patients from whom *N. meningitidis* were isolated ranged broadly from 1 to 76 years; although the ages of IMD patients ranged from 1 to 64 years, the age range for those who worked in military service was limited to 20 and 21 years.

Among the 19 isolates recovered from blood or CSF, the most common serogroup was serogroup B (7/19, 37%). All five isolates belonging to serogroup W were recovered from the blood or CSF of IMD patients who were involved in military service. Although serogroup W was no longer isolated after initiation of a vaccine programme (MenACWY-CRM, Menveo, GlaxoSmithKline) for military trainees, serogroup B caused meningitis at an army recruit training centre in 2015, two years after vaccination was introduced in 2013. Serogroup C was only found in IMD patients, similar to serogroup W. Serogroup E and the non-typeable serogroup were found since 2013. The six isolates recovered from sputum or the throat showed diverse serogroups: B, two isolates; Y, two isolates; E, one isolate; and non-typeable, one isolate.

In MLST analysis, 14 sequence types were found (Table 1), and 11 types were associated with IMD. Among the seven isolates belonging to serogroup B recovered from IMD patients, three isolates showed ST-3091 consistent with clonal complex (cc) 269, but the combinations of the FetA type and PorA types were different from each other: B:P1.20, 23-2:F3-6:ST-3091(cc269); B:P1.19, 15:F3-68:ST-3091(cc269); and B:P1.19-2, 15:F3-6:ST-3091(cc269). Other serogroup B isolates recovered from blood and/or CSF belonged to cc35 (1), cc41/44 (1), cc60 (1), and cc162 (1). The two serogroup B isolates found in sputum and the throat belonged to cc41/44 (B:P1.22, 15:F3-6:ST-2136) and cc269 (B:P1.19, 15:F3-6:ST3091). All isolates belonging to W showed the same molecular epidemiologic characteristics (W:P1.5-1, 2-2:F3-9:ST-8912). ST-8912 was a very independent sequence type that was not assigned to any clonal complex in eBurst analysis. The four isolates in serogroup C belonged to two clonal complexes (cc11, cc32). Three isolates showed the same FetA type and PorA types and differed in two loci: C:P1.22, 14-6:F1-7:ST-11278(cc32) and C:P1.22, 14-6:F1-7:ST-12771(cc32).

**Table 1:** Summary of epidemiologic data of *N. meningitidis* isolated in South Korea from 2010 to 2016.

Diagnosis	Year	Sex	Age	Specimen	Military service	Serogroups	Clonal complex	Sequence type	FetA	PorA VR1	PorA VR2
IMD	2010	M	14	Blood		B	ST-35 complex	35	F4-1	22-1	14
IMD	2010	F	6	Blood		B	ST-60 complex	3014	F1-7	5	2
IMD	2010	M	14	Blood		B	ST-269 complex	3091	F3-6	20	23-2
IMD	2013	F	64	CSF		B	ST-269 complex	3091	F3-68	19	15
IMD	2014	M	1	CSF		B	ST-269 complex	3091	F3-6	19-2	15
IMD	2015	M	20	CSF	O	B	ST-41/44 complex/Lineage 3	44	F4-27	12-1	1
IMD	2016	F	44	Blood		B	ST-162 complex	162	F5-9	20	NA
IMD	2013	F	22	Blood		C	ST-11 complex/ET-37 complex	11	F3-6	5-1	10-8
IMD	2013	F	39	CSF		C	ST-32 complex/ET-5 complex	12771	F1-7	22	14-6
IMD	2015	M	20	Blood		C	ST-32 complex/ET-5 complex	11278	F1-7	22	14-6
IMD	2016	M	20	Blood		C	ST-32 complex/ET-5 complex	11278	F1-7	22	14-6
IMD	2011	M	20	CSF	O	W	NA	8912	F3-9	5-1	2-2
IMD	2011	M	20	Blood	O	W	NA	8912	F3-9	5-1	2-2
IMD	2012	M	21	CSF	O	W	NA	8912	F3-9	5-1	2-2
IMD	2012	M	20	CSF	O	W	NA	8912	F3-9	5-1	2-2
IMD	2012	M	20	Blood	O	W	NA	8912	F3-9	5-1	2-2
IMD	2014	F	54	Blood		E	ST-178 complex	178	F5-5	19	15
IMD	2015	M	16	CSF		E	ST-41/44 complex/Lineage 3	44	F1-7	7-2	2-2
IMD	2015	M	20	CSF		E	ST-23 complex/Cluster A3	1655	F4-1	5-1	10-1
Pneumonia	2012	M	69	Sputum		B	ST-41/44 complex/Lineage 3	2136	F3-6	22	14
Pneumonia	2012	F	37	Sputum		Y	NA	12770	F4-1	5-1	2-2
Pneumonia	2014	F	76	Sputum		E	ST-178 complex	178	F5-28	19	15
Pneumonia	2013	M	51	Sputum		NT	ST-41/44 complex/Lineage 3	44	F1-7	NA	13-2
Carrier	2010	M	46	Throat		Y	NA	12768	F5-8	5-1	2-2
Carrier	2016	M	27	Throat		B	ST-269 complex	3091	F3-6	19	15

*Abbreviations: MLST, multilocus sequence typing; CC, clonal complex; NA, not available; URI, upper respiratory infection.

The three serogroup E isolates found in IMD patients showed different clonal complexes, E:P1.19, 15:F5-5:ST-178(cc178), E:P1.7-2, 2-2:F1-7:ST-44(cc41/44), and E:P1.5-1, 10-1:F4-1:ST-1655(cc23), and the one isolate from sputum showed the same sequence type and PorA type as one blood isolate but a different FetA type (E:P1.19, 15:F5-28:ST-178(cc178)). Serogroup Y was only found in sputum and throat isolates, and both isolates showed the same PorA type but different sequence and FetA types (Y:P1.5-1, 2-2:F4-1:ST-12770, Y:P1.5-1, 2-2:F5-8:ST-12768). The serogroup of one isolate belonging to ST-44 could not be determined by two molecular methods and latex agglutination.

**Figure 2:**
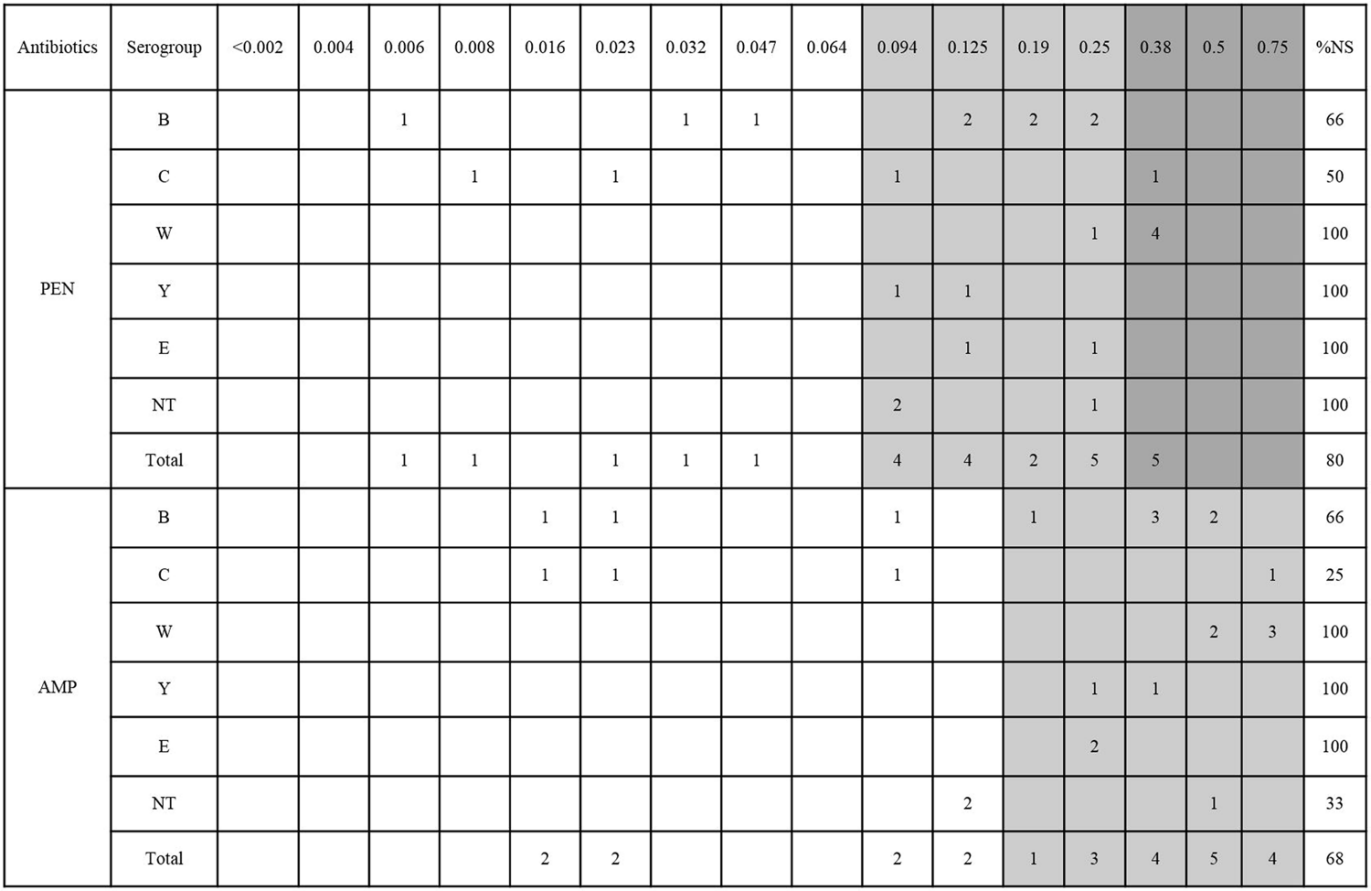
Distributions of MIC values against penicillin and ampicillin according to serogroup (shaded areas indicate intermediate and resistant ranges). Abbreviations: AMP, ampicillin; NS, non-susceptible; PEN, penicillin.

The antimicrobial susceptibility determined by Etest is summarized in Table 2. All of the isolates showed 100% susceptibility to ceftriaxone, meropenem, ciprofloxacin, minocycline and rifampin. Ceftriaxone and meropenem showed MIC ranges of ≤0.002–0.004 µg/mL and 0.002–0.064 µg/mL, respectively. Overall the MIC_50_ and MIC_90_ of ceftriaxone were ≤0.002 µg/mL and 0.003 µg/mL, and those of meropenem were 0.023 µg/mL and 0.047 µg/mL, respectively. The rates of isolates susceptible to penicillin and ampicillin were 20% and 32%, and the MIC ranges were 0.006–0.38 µg/mL and 0.016–0.75 µg/mL, respectively. Five isolates (20%) showed penicillin MIC of values of 0.38 µg/mL, which was higher than the intermediate breakpoints, and four of them belonged to serogroup W, while one was in serogroup C (Fig. 2). Isolates belonging to serogroup W also showed high MIC values against ampicillin (MIC range 0.5–0.75 µg/mL, intermediate).

**Table 2:** Antimicrobial susceptibility of *N. meningitidis* isolated in South Korea from 2010 to 2016 (n = 25).

Antibiotics	Distribution of MIC (µg/mL)			Antimicrobial susceptibility (%)		
	Range	MIC_50_	MIC_90_	Susceptible	Intermediate	Resistant
Penicillin	0.006–0.38	0.125	0.38	22	56	22
Ampicillin	0.016–0.75	0.25	0.75	30	70	0
Ceftriaxone	≤0.002–0.004	≤0.002	0.003	100	0	0
Meropenem	0.002–0.064	0.023	0.047	100	0	0
Ciprofloxacin	0.002–0.006	0.003	0.004	100	0	0
Minocycline	0.047–0.38	0.19	0.25	100	0	0
Rifampin	0.003–0.125	0.012	0.023	100	0	0

## Discussion

The prevalence of IMD may vary widely in different regions and countries. In the United States, the CDC reported that the prevalence of meningitis and bacteremia due to *N. meningitidis* was 0.16 cases/100,000 individuals; in 2015, 12.5% of these infected patients died (the mortality of meningitis was 9.1% and of sepsis 20%)^3^. In Europe, the mean prevalence of IMD was found to be 0.5 cases/100,000 individuals (range 0.2–3.1 cases/100,000) in 2014 by the European CDC^8^. In South Korea, all cases of IMD must be reported to the Korean CDC by law for infectious disease prevention and control, and the prevalence of meningococcal meningitis in the country was recently shown to be very low since 2010. A total of 46 cases of meningococcal infection confirmed by culture, serologic methods, or molecular tests were reported from 2010 to 2016 (6.57 cases per year)^9^, and these cases also represent the total IMD cases in South Korea during the course of this study. The recovery rate of culturable *N. meningitidis* from patients who are suspected to have IMD is generally low in other reports^10^. According to Bronska *et al*., only 35% of CSF and 39% of blood samples were culture positive among 37 patients with laboratory-confirmed IMD. In another study in England, which evaluated the value of PCR testing for IMD, the positive rate of culture was 42.9% among 1,924 IMD cases confirmed by PCR and culture^11^. In this study, we collected 19 isolates recovered from blood or CSF and six isolates from sputum and the throat. The number of isolates (19/46, 41.3%) collected was adequate when the low recovery rate of culture is considered. Although the prevalence of IMD was very low in South Korea, IMD also occurred, and a small outbreak of serogroup Y belonging to cc23 (P1.5-1, 2-2) was reported during 2002 and 2003^12^. In addition, Kim *et al*. reported that 13.2% (n = 92/608) of CSF specimens collected in 1999 and 2001 were positive for *N. meningitidis*, and it was the most prevalent pathogen in prospective population-based surveillance for invasive bacterial meningitis performed in children aged <5 years^13^. Furthermore, the high seroprevalence in the South Korean population between 11 and 55 years of age suggests the possibility of an underestimation of the IMD burdens in South Korea^14,15^. Such an underestimation may occur for several reasons, such as a high antibiotics prescription rate in South Korea, difficulties in sampling and testing at primary clinics, and the fastidious nature of *N. meningitidis*. Therefore, surveillance for meningococcal disease to determine its epidemiologic characteristics should be performed.

Determination of the serogroup of epidemic *N. meningitidis* isolates causing invasive infections is very important to establish a vaccine programme for public health. Vaccines currently available around the world include those based on polysaccharides, polysaccharide conjugates, recombinant protein, and outer membrane vesicles. Among them, quadrivalent conjugated meningococcal vaccines for serogroups A, C, Y and W are widely inoculated in many countries. Serogroup B vaccines have been introduced in infants in the UK National immunization programme in 2015, in Ireland and in regional programmes in Italy^16,17^. These vaccines have also been used for outbreak control in the Saguenay Lac-Saint-Jean region in Canada as well for other outbreak control in US universities and elsewhere^18^.

Among the 71 cases of meningitis and sepsis caused by *N. meningitidis* in the United States in 2015, 44 cases were tested to determine the serogroups, and serogroup B (41%) was the most prevalent^2^. In Europe, serogroup B was also the most dominant and mainly prevalent in infants but decreased in prevalence with age^8^. Since national immunization programmes with MenC conjugate vaccines were introduced in the UK in 1999 and then progressively across Europe, IMD due to serogroup C decreased in this region^19^. In contrast to developed countries, serogroup A was the most common serogroup in the meningitis belt of the sub-Saharan region^19,20^; however, it became rare due to the introduction of MenAfrivac, while outbreaks caused by serogroups C, W, and X were also reported sporadically^21,22^. According to a previous report^23^, serogroups X and Y were the most prevalent in CSF samples of paediatric meningitis patients from 1999 to 2002 in South Korea. However, the most prevalent serogroup became serogroup B from 2010 to 2016, which was isolated every year except for 2011 in our study. In molecular epidemiologic analysis, cc269 seemed to be the main clonal complex among serogroup B. It was reported that serogroup B (cc32, cc41/44, and cc269) included the endemic strains responsible for sporadic infections across Europe^24^ and was regarded as an emerging clone in Canada and the United States^25,26^. Serogroup B bearing P1.19,15, which may belong to cc269, was also found among freshman in a university dormitory in South Korea^27^.

It was surprising that a small outbreak of W occurred in 2011 and 2012 in a military training camp in South Korea. It has been suspected that the W outbreak occurred because all of these isolates share the same MLST, FetA type and PorA types (W:P1.5-1, 2-2:F3-9:ST-8912), although the isolated regions were very dispersed around the nation (Seoul 1, Gyeonggi 1, Busan 1, and Daejeon 2). ST-8912 showed a very independent relationship with the major clonal complex in eBurst analysis. It is not clear why W:P1.5-1, 2-2:F3-9:ST-8912 caused an outbreak among military trainees and from where this clone evolved, so further study is required. It is well known that new military trainees can be highly susceptible to IMD, so meningococcal vaccination is recommended for this group. However, meningococcal vaccines were not introduced until late 2012 in South Korea, and no additional W infections were identified after initiation of a meningococcal vaccine programme. However, serogroup W belonging to P1.5-1, 2-2 (ST not available) was also reported among freshmen in a university dormitory, and the possibility of an outbreak of the W clone remains^27^. In some cases, introduction of a vaccine can be associated with shifting of the serotype or serogroup and emergence of new serogroups. Differences in the serogroup distribution are also found in European countries^28^. The emergence of hypervirulent serogroup C (ST-11) led to the introduction of a meningococcal conjugate vaccine programme for serogroup C in Europe^29^, and the reduced proportion of serogroup C caused the proportion of serogroup B to increase^8,28^. It remains unclear if the emergence of one serogroup B infection belonging to B:P1.12-1, 1:F4-27:ST-44(cc41/44) in military trainees in 2015 resulted from the serogroup shifting due to vaccination, but serogroup B belonging to cc41/44 could be found in healthy South Korean adolescents in 2015^30^. Further surveillance and the introduction of a vaccine for serogroup B may be required when the prevalence of serogroup B in both patients and carriers is considered in South Korea. It is also possible that the emergence of serogroup E and serogroup non-typeable isolates may be associated with the partial application of a vaccine programme, even though serogroup E and non-groupable isolates tend to be common in asymptomatic carriage in healthy adolescents (E 12.2% and non-typeable 30.6)^30^. In our study, two IMD cases caused by serogroup E occurred in cancer patients who were immunocompromised.

Three serogroup C isolates in 2013, 2015, and 2016 share the same FetA and PorA types and belong to the same cc32, showing a two-allele difference. This clone was already reported in a study that screened university freshmen in 2009^27^. Even though the vaccine programme for military trainees started in late 2012^31^, the quadrivalent meningococcal vaccine is still not included in the mandatory vaccine programme for the general population and is only part of the programme for military service and high-risk patients in South Korea. It is possible that the persistence and dissemination of serogroup C cc32 in years 2013 and 2016 were associated with the absence of a National Immunization Program except for the military service; therefore, an expansion of the programme may be needed in order to consider serogroup C as the second most commonly carried type in healthy adolescents in 2015^30^.

The *N. meningitidis* isolates showed susceptibility to most antibiotics except for penicillin and ampicillin. Penicillin is regarded as the primary drug treatment in many countries when meningococcal infection is suspected. However, in our study, 78% and 70% of *N. meningitidis* isolates showed non-susceptibility to penicillin and ampicillin, respectively, and 22% of isolates were resistant to penicillin (MIC 0.38 μg/mL). The isolates that showed non-susceptibility to penicillin and ampicillin were W clones. It was reported that modification of penicillin binding protein 2 results in the acquisition of resistance to penicillin, and mosaicism can confer cefixime resistance similar to *N. gonorrhoeae*^7,32,33^. *N. meningitidis* isolates with decreased ciprofloxacin susceptibility were reported in 2016^34^, but all isolates were susceptible to ciprofloxacin, with the highest MIC being 0.006 µg/mL in our study.

In conclusion, the most prevalent serogroup of *N. meningitidis* isolates in South Korea changed from serogroup X and Y from 1999 to 2001 to serogroup B from 2010 to 2016, and additional introduction of a vaccine for serogroup B may be required. Serogroup W (W:P1.5-1, 2-2:F3-9:ST-8912), which was responsible for the small military outbreak, is unique and very independent from major clonal complexes. Antimicrobial susceptibilities to ceftriaxone, meropenem and ciprofloxacin remained, but most of the isolates were non-susceptible to penicillin and ampicillin. Further surveillance of serogroup changes and antimicrobial resistance is needed to control IMD in South Korea.

## Methods

From April 2010 to November 2016, a total of 25 *N. meningitidis* isolates were collected in collaboration with the Korean Society of Clinical Microbiology (KSCM). The KSCM is an academic society consisting of clinical microbiologists who primarily manage clinical microbiology laboratories in hospitals, commercial laboratories, and national institutes in South Korea. All hospitals with members of the KSCM were regularly notified of the collection of *N. meningitidis* isolated from clinical specimens. Isolate collection was promoted by regular e-mail notifications from 2010 to 2016. When *N. meningitidis* was isolated from clinical specimens, it was transferred to the Research Institute of Bacterial Resistance at Yonsei University College of Medicine. The collected isolates were stored in a deep freezer at −70 °C or below prior to analysis. The study design and protocol were reviewed and approved by the Institutional Review Board of Severance Hospital in the Yonsei University health system (IRB No. 4-2010-0567).

The collected isolates were identified using a Vitek-2 system (bioMerieux, Marcy-l’Etoile, France) and a Microflex LT system (Bruker Daltonics, Bremen, Germany). If needed, 16S rRNA amplification and sequencing were performed for identification. The 16S rRNA gene was amplified using primers, as suggested by the Clinical and Laboratory Standards Institute^35^, and sequenced at a commercial laboratory (Macrogen, Seoul, South Korea). Data were analysed using either the GenBank database (http://www.ncbi.nlm.nih.gov/genebank/) or EzBioCloud service (https://www.ezbiocloud.net/identify). Serogroups were screened with two molecular methods and confirmed by slide latex agglutination. Conventional multiplex PCR was carried out for the *siaD* gene for serogroups B, C, W, and Y and the *orf-2* of a gene cassette for serogroup A^36^. Bacterial colonies were suspended in 500 μL sterile distilled water for DNA extraction, and the DNA concentration was estimated using a spectrophotometer. The DNA amplification process consisted of 35 cycles of denaturation at 92 °C for 40 s, annealing at 55 °C for 30 s, and polymerization at 72 °C for 20 s. The size of the amplicon was measured by electrophoresis and compared with quality control strains for confirmation of serogroup A (CCUG3269), B (CCUG3270), and C (CCUG3271). Amplicons corresponding to serogroups W and Y were sequenced by Sanger methods and analysed. The isolate for which the serogroup could not be determined was tested by another molecular method reported by Bennett *et al*.^37^. Multiplex PCRs for the *ctrA* gene, serogroup-specific region (X, E, Z and H), and *porA* gene were amplified, and the product was sequenced for confirmation (Macrogen).

Slide latex agglutination was also performed to confirm the serogroup using BD Difco™ Neisseria meningitidis antisera (Becton Dickinson, Sparks, MD, USA). After scraping and suspending several colonies of pure cultured *N. meningitidis*, one drop of colony suspension and antisera for each serogroup was mixed with each of the test cards (serogroups A, B, C, D, E, W, X, Y, and Z). Agglutination was regarded as positive, and the result was compared with the result of multiplex PCR serogrouping. Multilocus sequence typing (MLST), ferric enterobactin transport protein A (FetA) typing, and variable regions (VR) in Porin A (PorA) typing were performed to determine the epidemiologic characteristics in all isolates as described in http://neisseria.org/.

The antimicrobial susceptibility was determined using Etest strips (bioMerieux). The minimum inhibitory concentrations of penicillin, ampicillin, ceftriaxone, meropenem, minocycline, ciprofloxacin, and rifampin were measured after incubation for 20–24 hours at 35 °C in 5% CO_2_ with Mueller-Hinton agar with 5% sheep blood, and the results were interpreted according to the Clinical Laboratory Standards and Institute (CLSI) guidelines^38^.

### Data availability

The datasets generated and/or analysed during the current study are available from the corresponding author upon reasonable request.
